# Effectiveness of an add-on brief group behavioral activation treatment for depression in psychiatric care: a randomized clinical trial

**DOI:** 10.3389/fpsyt.2024.1284363

**Published:** 2024-04-30

**Authors:** Riikka Haakana, Tom Rosenström, Lauri Parkkinen, Martti T. Tuomisto, Erkki Isometsä

**Affiliations:** ^1^ Department of Psychiatry, University of Helsinki and Helsinki University Hospital, Helsinki, Finland; ^2^ Department of Psychology and Logopedics, Faculty of Medicine, University of Helsinki, Helsinki, Finland; ^3^ Faculty of Social Sciences (Psychology), Tampere University, Tampere, Finland

**Keywords:** depression, randomized clinical trial, group therapy, behavioral activation (BA), peer support (PS)

## Abstract

**Objective:**

Behavioral activation (BA) is an effective treatment for depression. We investigated the effectiveness of add-on group-format BA and peer support (PS) with treatment as usual (TAU) in a registered randomized clinical trial in psychiatric outpatient settings (ISRCTN10647845).

**Methods:**

Adult outpatients (N = 140) with major depressive disorder (MDD) and Patient Health Questionnaire (PHQ-9) score ≥10 were randomized into a) group BA, consisting of eight 90-minute weekly group sessions plus TAU; b) group PS, including eight 90-minute weekly group sessions plus TAU; or c) TAU alone. The primary outcome was a within-individual change in PHQ-9 score between baseline and 8 weeks. Secondary outcomes were 1) response, 2) remission, and 3) functional impairment at 8 weeks, plus 4) change in PHQ-9 at 6 months.

**Results:**

Of the randomized patients, 100 (71.4%) completed treatments, including 29/45 (64.4%) patients in the BA group, 39/49 (79.6%) in the PS group, and 32/46 (69.6%) in the TAU group. By 8 weeks, PHQ-9 scores declined most in the TAU group [BA −0.28 (95% CI −2.48, 1.92), PS −0.58 (−2.09, 0.94) vs. TAU −3.32 (−5.21, −1.44); group-difference test, *p* = 0.034]. The secondary outcomes in the BA or PS arms did not significantly differ from those in TAU. Videotaped sessions revealed marked variation in briefly trained therapists’ adherence to the treatment manual.

**Conclusions:**

In this randomized trial, the effectiveness of treatments with the added BA and PS groups did not exceed that of TAU alone. The preconditions in which brief BA or PS group interventions benefit outpatients with depression in psychiatric settings warrant critical investigation.

## Introduction

Depression is a central public health problem and a leading cause of disability worldwide (1). There is a great need for effective brief psychotherapeutic interventions for depression in healthcare. Organizing treatment in groups can improve the cost-effectiveness and scalability of treatments by allowing more patients to be concurrently treated.

Behavioral activation (BA) for depression is an intervention in which patients learn techniques to monitor their mood, daily activities, and the association between them. In BA, patients will also learn how to develop a plan to increase the number of pleasant activities and positive interactions with their environment. Meta-analyses of studies of BA in the treatment of depression have yielded evidence of effectiveness, although high-quality studies are still needed (2, 3). In a meta-analytic dismantling study, BA was the only independently beneficial component of Internet cognitive behavioral therapies (4). A meta-analysis found BA to be effective also in group format (5). A significant advantage of BA is that it does not require years of training or complex skills from the therapist, and mental health professionals without previous experience in BA can deliver effective treatment (6). A large multicenter trial comparing the effectiveness of individual BAs provided by junior professionals with the cognitive therapy delivered by psychotherapists in primary healthcare settings found BA to be equally effective but more cost-efficient (7).

Significant differences in the effectiveness of most common psychotherapies for depression have not been noted (8). Based on observational evidence, psychological interventions will likely be effective as part of routine practice (9). However, given that less than half of patients respond to treatment (defined as ≥50% symptom reduction), more effective treatments and treatment strategies are needed (10). To enhance the development of effective psychotherapies, studying postulated mediators and moderators of treatment effects advances knowledge of the mechanisms of change (11). Alleviation of experiential avoidance and anhedonia (missing positive reinforcement) has been seen as a putative mediator of the clinical effects of BA (12), although evidence on the mediators of BA remains weak (13).

The purpose of peer support (PS) is to provide non-professional support for patients with the same health problems or stressors, with experienced peers offering support for novice peers. Peer support positively affects depression recovery (14) and is a cost-effective form of adjunctive treatment. Whether or not PS groups benefit patients with depression in Finnish psychiatric care remains unknown. Furthermore, due to the lack of specific theoretically founded therapist interventions, a PS group may also serve as an acceptable non-specific control intervention when evaluating group interventions expected to have a specific, theoretically founded efficacy.

The aim of this study was to investigate whether a brief training of psychiatric professionals and implementation of added low-cost group treatments can improve the symptoms of depressive patients. We hypothesized that 1) group BA by briefly trained professionals combined with treatment as usual is more effective than the PS group combined with treatment as usual (TAU) or TAU alone, 2) PS treatment combined with TAU is more effective than TAU alone, and 3) effectiveness of BA is partly mediated by patients’ behavioral activity and consequent reduction in experiental avoidance and anhedonia.

## Methods

### Study setting and design

The study was a randomized, three-arm, parallel-group clinical trial, and it was carried out in Finland at Helsinki University Hospital (HUH), Department of Psychiatry, Mood Disorders division outpatient clinics. It had been registered as a clinical trial (ISRCTN10647845). The study was approved by the Ethics Committee of the Helsinki and Uusimaa Hospital District (HUS) and granted a research permit by the HUH Department of Psychiatry.

The patients enrolled in the study were recruited from outpatient clinics in the cities of Espoo and Vantaa. The attending professionals requested the consent of patients to participate in the study. Eligible patients were informed about the study, and those volunteering gave written informed consent. The interventions and data collection were carried out between October 2016 and June 2017.

### Participants

Patients who were adults (18–65 years), fluent in Finnish, fulfilled diagnostic criteria of DSM-IV major depressive disorder (MDD), and had a current Patient Health Questionnaire (PHQ-9) score ≥10 were eligible for the study. Exclusion criteria were chronic MDD (uninterrupted duration of major depressive episode >2 years), psychotic features, principal diagnoses of borderline personality disorder or substance use disorder, imminent threat of suicide, need for psychiatric hospitalization, any illness or symptom hampering participation in the treatments, and other ongoing weekly psychotherapy.

Participants met the criteria of major depressive disorder according to the Diagnostic and Statistical Manual of Mental Disorders, 4th ed. (DSM-IV) (15), and had a PHQ-9 (16) score ≥10. DSM-IV diagnoses were based on the Structured Clinical Interview for the DSM-IV Axis I Disorders (SCID-I-CV) (17) or the Mini-International Neuropsychiatric Interview (MINI) (18). The diagnostic process was implemented as part of the clinical work at the outpatient clinics.

**Figure 1:**
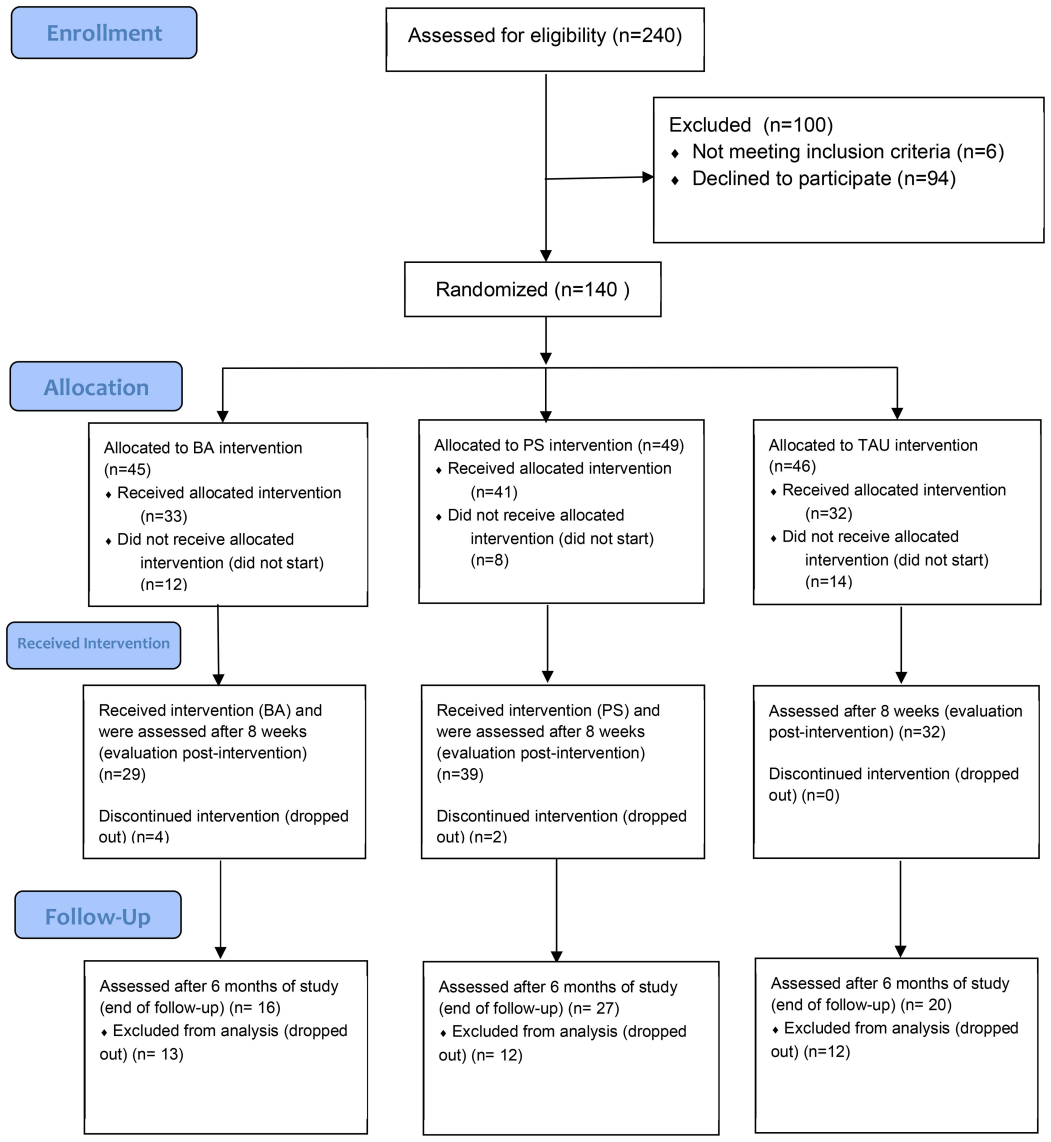
Flowchart.

**Figure 1** presents the flow of participants throughout the study. Willingness to participate in the study was enquired of 240 depressive patients; 94 patients (39.2%) declined to participate. Five consenting participants were excluded because of a low score (<10) on the PHQ-9, and one participant was excluded because of failure to meet the criteria for MDD. Altogether, 140 patients met the inclusion criteria and were randomized into treatment groups.

Participants’ mean age was 38 years (SD 12, range 20–59 years), and 63% were women and 37% were men. **Table 1** shows their demographic characteristics by treatment arm.

**Table 1:** Demographic characteristics in treatment groups.

		BA n = 33 (%)	PS n = 41 (%)	TAU n = 32 (%)	Total N = 106 (%)
**Sex**	Female	23 (69.7)	27 (65.9)	17 (53.1)	67 (63.2)
	Male	10 (30.3)	14 (34.1)	15 (46.9)	39 (36.8)
**Age**	Mean age in years (s.d.)	39.2 (11.6)	40.2 (13.0)	35.4 (12.1)	38.3 (12.4)
**Marital status**	Single	14 (42.4)	20 (48.8)	14 (43.8)	48 (45.3)
	Cohabiting	5 (15.2)	5 (12.2)	6 (18.8)	16 (15.1)
	Married	6 (18.2)	10 (24.4)	6 (18.8)	22 (20.8)
	Divorced/separated	8 (24.2)	6 (14.6)	5 (15.6)	19 (18.0)
	Unknown			1 (3.1)	1 (0.9)
**Living alone**	Living alone	13 (39.4)	14 (34.1)	10 (31.3)	37 (34.9)
	Not living alone	20 (60.6)	27 (65.9)	22 (68.8)	69 (65.1)
**Education**	Some comprehensive school	2 (6.1)	1 (2.4)	1 (3.1)	4 (3.8)
	Comprehensive school	12 (36.)	17 (41.5)	13 (40.6)	42 (39.6)
	Some secondary school	8 (24.2)	5 (12.2)	4 (12.5)	17 (16.0)
	Matriculation examination	11 (33.3)	18 (43.9)	13 (40.6)	42 (39.6)
Vocational and professional education					
	University	5 (15.2)	4 (9.8)	2 (6.3)	11 (10.4)
	University of applied sciences	6 (18.2)	11 (26.8)	4 (12.5)	21 (19.8)
	Vocational school	7 (21.2)	11 (26.8)	7 (21.9)	25 (23.6)
	Apprenticeship training	1 (3.0)	0 (0)	2 (6.3)	3 (2.8)
	Lacking or unknown	14 (42.4)	15 (36.6)	17 (53.1)	46 (43.4)

BA, behavioral activation; PS, peer support; TAU, treatment as usual.

### Randomization

Randomization was conducted by the last author (E.I.) using the Research Randomizer program (www.randomizer.org), stratified into four lines, one for each outpatient clinic (Matinkylä and Leppävaara in Espoo; Myyrmäki and Tikkurila in Vantaa). The interviewing professionals were blind to the patients’ allocation in randomization, and the patients were only afterward informed by telephone about the group to which they were assigned.

Thirty-three patients started the study in the BA intervention, 41 in the PS intervention, and 32 in the TAU intervention. Thirty-four randomized participants (24.3%) did not start the treatment. After the onset of the intervention, 12 participants (11.3% of the 106 who started) dropped out (**Figure 1**).

Twelve BA groups and 12 PS groups were organized in four outpatient clinics (two in Vantaa and two in Espoo). All participants were evaluated 1) before treatment, 2) after treatment at 8 weeks, and 3) at the 6-month follow-up.

### Study measures

At the beginning of the study, we gathered sociodemographic information (**Table 1**). All participants also completed the following questionnaires: 1) Sheehan Disability Scale (19), which is a self-report tool that assesses functional impairment in work/school, social life, and family life by three scales scored 0–10; 2) Alcohol Use Disorders Identification Test (Audit) (20), which is a 10-item questionnaire with 0–4 scale on each item and provides a versatile picture of the respondent’s alcohol consumption; 3) S5 (21) is a 60-item personality questionnaire; 4) Perceived Social Support—Self-Report (PSSS-R) (22) is a 12-item (scored 1–5) questionnaire of subjective social support; and 5) Patient Health Questionnaire (PHQ) assesses the severity of depression. PHQ-9 (16) is the nine-item depression module of the PRIME-MD diagnostic instrument for common mental disorders. It scores each of the DSM-IV criteria from “0” (not at all) to “3” (nearly every day) over the last two weeks.

Before each PS and BA group meeting, the participants completed the following questionnaires to monitor changes weekly: 1) Beck Depression Inventory (BDI) (23) contains 21 questions measuring symptoms of depression. 2) The Snaith-Hamilton Pleasure Scale (SHAPS) (24) is a 14-item instrument that measures anhedonia. Each item has four response categories (1 = definitely agree, 2 = agree, 3 = disagree, and 4 = definitely disagree), and a higher score indicates strong anhedonia. 3) Acceptance and Action Questionnaire (AAQ-2) (25) assesses experiential avoidance, acceptance, and psychological inflexibility. It is a seven-item questionnaire. 4) Behavioral Activation for Depression Scale (BADS) (26) measures the frequency of activation, escape, and avoidance. We used the nine-item version, in which five questions measure the amount of activation and four questions measure the experiential avoidance. It was used only in the BA groups.

After treatments at 8 weeks, all participants completed the PHQ-9, Sheehan Disability Scale, BDI, SHAPS, and AAQ-2. The 6-month follow-up was carried out using mailed BDI and PHQ-9 questionnaires.

### Interventions

#### Treatment as usual

The participants in the control group (TAU) received their usual treatment for depression as outpatients. The treatment consisted mainly of antidepressant pharmacotherapy and low-intensity psychotherapeutic support provided by professionals (psychiatric nurses, psychologists, social workers, or occupational therapists). The number of visits was not predefined.

In addition to the usual treatment, the PS and BA group participants received weekly group-based treatments for 8 weeks.

#### Peer support group

The PS treatment included eight 90-min group sessions once a week. Our goal was to form groups of eight people, but due to recruitment difficulties, the size of the groups varied between one and eight. The groups were facilitated by an expert with experience in having recovered from depression, and they received brief training for the task from the Finnish Central Association for Mental Health. One psychiatric nurse participated in each PS group session but did not have an active facilitator role. Before each group session, participants were asked to complete BDI, SHAPS, and AAQ-2 questionnaires. The aim of the PS group was to increase social PS, involvement, activity, and opportunities.

#### Behavioral activation group

The BA group treatment consisted of eight 90-minute weekly meetings. The size of the groups also varied between one and eight people. Before each therapy session, participants were asked to complete BDI, SHAPS, AAQ-2, and BADS questionnaires. Two therapists delivered each BA group.

All therapists had long experience in psychiatric care, and they were psychologists (2), nurses (9), social workers (1), or occupational therapists (4) by profession. The group leaders received training on BA (2 × 4 hours) and 2 hours of supervision four times in the 8-week group. The training followed the principles of BA presented by Kanter et al. (12) and focused on the rationale and skills required to deliver an eight-session protocol of BA for depression. Most of the group leaders had experience in leading a BA group from the pilot phase that was organized before the start of the study.

Specific techniques in the BA group were self-monitoring, identifying depressed behaviors, developing alternative goal-orientated behaviors, and scheduling meaningful activation. Functional analysis of behavior was used to examine the role and occurrence of avoidance and rumination. The goal of BA was to re-engage participants with sources of positive reinforcement.

#### Assessment of treatment adherence by therapists

We assessed the quality of and adherence to BA treatment using videotapes of the therapy sessions. All BA group sessions (92 group sessions) were recorded, and the recordings were randomized. An independent expert in BA rated a random sample (10%, 12 sessions) of recordings for competence using a scale based on the revised cognitive therapy scale (CTS-R) (27). There were 14 factors to be evaluated, and the scale of the evaluation was 0–6 (0 = “absence of the feature, or highly inappropriate performance” to 6 = “excellent performance, or very good even in the face of patient difficulties”).

### Outcomes

#### Primary outcomes

The primary outcome was a change in an individual’s depression score as measured by the PHQ-9 at baseline and after the 8-week intervention or 8 weeks of TAU.

#### Secondary outcomes

The secondary outcomes were 1) treatment response, defined as ≥50% decline in PHQ-9 score from baseline to 8 weeks; 2) remission, defined as PHQ-9 score <5 at 8 weeks; 3) reduction in functional impairment, measured using the Sheehan Disability Scale Score at baseline and 8 weeks; and 4) lasting reduction in depression score, measured using the PHQ-9 at baseline and 6 months after the intervention.

### Statistical methods

We first describe our rationale for statistical power. Estimating from their **Table 2**, COBRA study (7) reported a non-paired (Cohen’s) standardized intention-to-treat change score of *d* = −1.6 for BA. In contrast, Melartin et al. (28) observed for TAU that 10% remitted in 8 weeks, corresponding to an average [Hamilton Depression Rating Scale (HAMD)] symptom score of 2.7 (s.d. 2.8, so ~97.5% of the remitted were estimated below score 8.2 at remission, i.e., below 2.7 + 1.96 × 2.8). Assuming their standard deviation at 8 weeks was the same as that at the baseline (i.e., 6.1), a 3-point reduction moves 10% of the baseline population below a possible remission threshold (for normally distributed symptom scores). Hence, we approximated an expected TAU effect size of −3/6.1, corresponding to non-paired *d* ≈ −0.5. It seemed likely that the effect difference of BA and TAU would be at least *d* = 0.5, as our crude observations above suggested a difference greater than *d* = 1. At the time, a difference of *d* = 0.5 appeared possible for the BA vs. PS comparison, too. A sample size of 85 per treatment arm delivers 90% statistical power for pairwise (t-test) comparisons under the effect *d* = 0.5. Under this effect and assuming a proportion mediated 0.5, 49 observations deliver 90% power for testing mediation in a simple structural equation model (for rejecting the null effect of the mediator on the outcome, whereas 43 observations suffice to reject the null effect on the mediator—these are only possibilities under the mediation model). With the estimated upper-limit requirement of 85 patients per arm and anticipating some follow-up attrition, we aimed at 96 patients per treatment arm (a total target of 288 patients). Recruitment difficulties and attrition were much higher than initially anticipated, however. Given our final and reduced sample sizes, power for the BA-TAU comparison on PHQ-9 change reduced to 45% and that for BA-PS to 46%.

**Table 2:** Per-protocol outcome measure sample sizes, means, and standard deviations by study phase and treatment arm.

		BA			PS			TAU			Total		
		Mean	N	SD	Mean	N	SD	Mean	N	SD	Mean	N	SD
***PHQ-9***	Baseline	16.7	33	4.6	16.9	41	5.4	18.1	32	5.4	17.2	106	5.2
	8 weeks	16.8	25	6.4	15.7	33	4.9	14.6	31	5.4	15.6	89	5.5
	6 months	12.4	16	7.7	12.4	27	5.6	14.1	20	6.7	12.9	63	6.5
***BDI***	Baseline	29.7	29	8.1	31.2	31	8.9	32.3	31	9.0	31.1	91	8.7
	8 weeks	29.3	26	12.1	28.3	35	9.4	28.4	29	8.5	28.7	90	9.9
	6 months	24.3	15	11.3	26.2	26	10.1	26.8	20	12.1	25.9	61	10.9
***SHAPS***	Baseline	4.8	30	3.7	6.7	31	4.2				5.8	61	4.0
	8 weeks	5.5	26	4.3	7.4	36	4.1				6.6	62	4.2
***AAQ-2***	Baseline	33.0	31	7.6	32.4	30	9.6				32.7	61	8.6
	8 weeks	35.9	26	8.5	32.6	35	9.7				34.0	61	9.3

BA, behavioral activation; PS, peer support; TAU, treatment as usual; PHQ-9, Patient Health Questionnaire; BDI, Beck Depression Inventory; SHAPS, Snaith-Hamilton Pleasure Scale; AAQ-2, Acceptance and Action Questionnaire.

We used analysis of variance (*F*-test) and two-sided *t*-tests to evaluate the effects of the treatment arm on change in symptom scores over the treatment and Fisher’s exact test for binary-valued variables. We additionally used Pearson’s correlation coefficient as a simple measure of association. In addition to those per-protocol analyses (n = 89 for PHQ-9; 17 had baseline but lacked follow-up data on it), we conducted a partial intention-to-treat analysis. As we had no data on the 34 patients who were randomized to treatment arms but did not start the study nor from post-dropout periods, our intention-to-treat analysis could only correct for after-baseline dropout by means of statistical imputation using baseline information (n = 106). Multiple imputations were conducted using chained equations and predictive mean matching (29, 30). We monitored 35 imputation chains to convergence. The imputation model used data on age, sex, number of major depressive episodes (MDEs), number of children, employment and cohabitation status, PSSS-R, treatment group, and PHQ-9 and BDI changes.

To verify the causal structure required for testing our mediation hypothesis, we modeled lagged effects with mixed-effect linear modeling to investigate the direction of causation (indexed by temporal precedence) between patients’ behavioral activity (BADS score) and depressive symptoms assessed with BDI (31–33).

## Results

**Table 2** summarizes raw sample sizes and central and dispersion values on all the measured scales by study phase and treatment arm. Depression scores, on average, did not significantly decrease from baseline to 8 weeks in the BA and PS treatment arms.

### Primary outcome

Change in depression scores in the PHQ-9 between baseline and after the 8-week intervention was our predefined primary outcome for per-protocol analyses, reported in **Table 3**. In our omnibus test, we found statistical differences in PHQ-9 score changes across the treatment arms (*F*_2,86 =_ 3.517, *p* = 0.034). Pairwise t-tests indicated a difference between the BA treatment group and TAU (*p* = 0.036), and between PS and TAU (*p* = 0.024), but not between BA and PS (*p* = 0.821). However, only the TAU group’s PHQ-9 score significantly improved (declined) during the treatment, whereas changes in the other two treatment arms were statistically non-significant (see confidence intervals in the first row of **Table 3**).

**Table 3:** Tests of primary and secondary outcomes.

Outcome statistic	Group-wise parameters			Corresponding cross-group tests			
	BA (*n*_ITT_ = 34)^‡^	PS (*n*_ITT_ = 41)	TAU (*n*_ITT_ = 32)	*p*-Value for any difference	*p*-Value for BA+TAU vs. TAU	*p*-Value for PS+TAU vs. TAU	*p*-Value for BA+TAU vs. PS+TAU
Primary outcome (per-protocol analysis)							
PHQ-9 score change (95% CI)	−0.28 (−2.48, 1.92)	−0.58 (−2.09, 0.94)	−3.32 (−5.21, −1.44)	0.034	0.036	0.024	0.821
*p*-Value for PHQ-9 change	0.795	0.445	<0.001				
Standardized PHQ-9 change, Hedge’s g	−0.29	−0.23	−0.65				
Primary outcome (partial ITT^†^ analysis)							
PHQ-9 score change (95% CI)	−0.53 (−2.4, 1.35)	−0.39 (−1.75, 0.98)	−3.28 (−5.05, −1.51)	0.019	0.034	0.009	0.903
*p*-Value for PHQ-9 change	0.580	0.579	<0.001				
Secondary outcomes (per-protocol analysis)							
Response (≥50% decline in PHQ-9)	3/22 (14%)	0/33 (0%)	3/28 (11%)	0.105	1	0.108	0.075
Remission, n (%) with PHQ-9 < 5 at week 8	1/24 (4%)	0/33 (0%)	0/31 (0%)	0.281	0.446	1	0.431
Sheehan disability score change (95% CI)							
Family score (95% CI)	−0.40 (−1.12, 0.32)	−0.21 (−0.89, 0.46)	−1.19 (−1.80, −0.58)	0.075	0.091	0.031	0.699
Work score (95% CI)	−0.40 (−1.05, 0.25)	−0.25 (−0.86, 0.36)	−0.68 (−1.28, −0.08)	0.581	0.524	0.312	0.731
Social life score (95% CI)	0.40 (−0.39, 1.19)	0.42 (−0.25, 1.10)	−1.00 (−1.47, −0.53)	0.002	0.003	0.001	0.962
Six-month PHQ-9 score change (95% CI)	−4.18 (−8.05, −0.32)	−3.70 (−6.21, −1.20)	−2.95 (−6.18, 0.28)	0.856	0.607	0.704	0.826
Other outcomes (per-protocol analysis)							
BDI score change (95% CI)	0.32 (−2.98, 3.61)	−1.31 (−3.64, 1.02)	−2.89 (−5.68, −0.10)	0.260	0.131	0.375	0.409
SHAPS score change (95% CI)	1.22 (−0.12, 2.56)	0.81 (−0.29, 1.92)					0.635
AAQ-2 score change (95% CI)	3.29 (0.29, 6.30)	−0.35 (−2.88, 2.20)					0.063
BADS score change (95% CI)	0.03 (−0.44, 0.49)						

p-Values are from an F-test of group differences or a Welch’s t-test (paired comparisons) for continuous-valued variables (sum scores) and from Fisher’s exact tests for binary variables (remission rates).

BA, behavioral activation; PS, peer support; TAU, treatment as usual; BDI, Beck Depression Inventory; SHAPS, Snaith-Hamilton Pleasure Scale; AAQ-2, Acceptance and Action Questionnaire-2; PHQ-9, Patient Health Questionnaire.

^†^Intention-to-treat analysis from a multiple imputed regression (NB: corrects only for dropout after the baseline and not for all missing patients we intended to treat).

^‡^Treatment-group sizes refer to imputed ITT data and missingness varies by outcome in per-protocol analyses (numbers of change-score respondents in the BA, PS, and TAU groups were 25, 33, and 31, respectively, for PHQ-9; 25, 33, and 31, respectively, for Sheehan Family score; 25, 32, and 31, respectively, for Sheehan Work score; 25, 33, and 31, respectively, for Sheehan Social life score; 16, 27, and 20, respectively, for 6-month change in PHQ-9; 22, 26, and 28, respectively, for BDI; 23, 27, and 0, respectively, for SHAPS; 24, 26, and 0, respectively, for AAQ-2; and 24, 0, and 0, respectively, for BADS).

Change in BDI scores was not a primary outcome, but we report it to allow comparison for evaluating the consistency of results. We did not find a significant difference in group averages in BDI change scores (*F*_2,73 =_ 1.373, *p* = 0.260).

As the change scores contained some missing data (cf. **Table 2**), we carried out a multiple imputation analysis to achieve a partial intention-to-treat analysis. The findings agreed with the per-protocol analyses (see **Table 3** for simple summaries). We further examined age- and sex-adjusted PHQ-9 change as an additional sensitivity analysis on the intention-to-treat, but this also indicated 2.97 points lesser symptom reduction for the BA arm than for the TAU arm (95% CI = 0.49, 5.45; *p* = 0.019). The corresponding number for the PS arm was 3.08 points (95% CI = 0.78, 5.38; *p* = 0.009).

### Secondary and other outcomes

**Table 3** also shows results for the secondary and other outcomes. Few patients responded or remitted with group differences being non-significant for 50% symptom decline (*p* = 0.109) as well as for the absolute criterion of PHQ-9 score <5 (*p* = 0.281). The findings for family-related, work-related, and social life disability paralleled the primary outcomes; only the TAU group showed statistically significant improvement from baseline (**Table 3**). However, differences between groups were consistently significant only for the social life disability. There were no significant group differences in anhedonia (SHAPS not measured in TAU). The AAQ-2 score increased significantly in BA but not in the PS group (not measured in TAU).

At the 6-month follow-up, the change in PHQ-9 score from baseline was statistically significant for the BA and PS groups, but not for the TAU group. The numerical change was most favorable for the BA group (**Table 3**), albeit the difference compared with the TAU group was non-significant (*p* = 0.607 for Welch’s t-test).

### Mediational role of behavioral activity

We investigated how participants’ active participation or behavioral activity (BADS score) predicted the BA group’s treatment response (PHQ change). We found suggestive evidence that (time-average) active participation was correlated with greater PHQ-9 reductions (*r* = 0.38; 95% CI = −0.67, 0.02; *p* = 0.060). Notably, active participation at the end (at week 8) was associated with better treatment response, i.e., with a reduction in PHQ-9 (*r* = −0.64; 95% CI = −0.83, −0.33; *p* < 0.001). However, this may have reflected more reverse causation than effects of active participation on treatment response; patients with lower BDI scores in the previous week increased in BADS score despite adjusting the previous week’s BADS score with a lagged effect of −0.03 points (95% CI = −0.06, −0.01; *p* = 0.003; fixed effects in a mixed-effect model with random intercepts for patients). In an opposite prediction model, there was no lagged effect of the BADS score on the BDI score after adjusting the lagged BDI score (lagged BADS coefficient of −0.19; 95% CI = −1.10, 0.72; *p* = 0.684). There was thus no evidence for the activity-mediated effect of BA on depression (no main effect of BA on depression plus evidence against the causal-direction assumptions of such a mediation model).

### Treatment adherence by therapists

An independent expert in BA rated a 10% random sample (12 sessions) of videotaped recordings using the CTS-R scale. Marked variation in therapists’ adherence to the manual was observed. Only six of 12 assessed group meetings (50%) were rated as good (4 points or more).

## Discussion

In this study, we investigated the effectiveness of group-format BA and PS added to the usual treatment of depression in psychiatric outpatient care settings at Helsinki University Hospital. The study was designed as a randomized, three-arm, parallel-group study. We hypothesized that the BA group treatment plus TAU would be more effective than the PS group treatment plus TAU or TAU alone. However, the findings of the primary and secondary outcomes of the study did not support the hypotheses. Patients in the BA or PS groups actually benefited less from their treatment than patients receiving usual care in terms of the primary outcome and social life disability secondary outcome. Rather than past behavioral activity predicting depression scores, we observed the reverse temporality with lagged depression predicting behavioral activity. Given the lack of treatment effect and the evidence for violated causal assumptions (relative to the mediation model), the mediation hypothesis was considered unsupported by the data. The study also demonstrates problems that can be encountered in research and implementation of brief psychotherapeutic group interventions in secondary-level psychiatric settings, as further discussed below.

The strengths of this study included being registered, controlled, randomized, and carried out in the usual treatment environment within psychiatric outpatient care. The numbers of randomized patients or patients completing treatment in the BA or TAU cells (140, 29, and 32) were at the high end of the range of previous studies (5). The brief training of therapists and the brief group-format interventions were deliberate methodological decisions made considering the potential for future scalability within psychiatric care. All study patients had been diagnosed with MDD using a structured interview (SCID-I or MINI). Further, all BA group sessions were videotaped, and an external expert assessed adherence to the principles of BA in 10% of randomly chosen sessions. It allowed critical evaluation of the treatment provided.

However, several methodological issues must be noted. First, we designed our study to compare the effectiveness of added-on BA and PS with TAU alone, rather than comparing standalone BA or PS interventions with TAU. Whether add-on BA or PS interfered with the TAU remains unknown. Second, recruiting patients to treatment groups in outpatient care settings was more difficult than expected, and the number of participants in the study remained markedly lower than the initial target size. This was due to multiple factors. Unexpectedly, difficulty judging whether a patient’s depression was chronic (uninterrupted over 2 years, excluded) or recurrent with interepisodic residual symptoms (included) turned out to be clinically difficult, and uncertainty reduced the number of potentially eligible patients assessed. Furthermore, 39% of eligible depressive patients declined to participate in the study, and approximately 24% of randomized patients failed to start their allocated treatment. Therefore, the sample size and the intervention (BA and PS) groups became smaller than planned and had two to four group members (one group only had one member). For comparison, the minimum group size for inclusion in the meta-analysis by Simmonds-Buckley et al. (5) was three patients. In contrast, the dropout rate from the intervention groups (range 9%–12%) was similar to that of other studies of group BA for depression (5). An imputation analysis somewhat mitigated the problem for those with baseline data. Third, as in almost all other group BA studies (5), our findings of change between baseline and end of treatment at 8 weeks represent completer rather than intention-to-treat analyses (imputations were unavailable for those without any baseline data). Fourth, all our outcomes were based on self-report. Fifth, we did not formally measure patient satisfaction, although the spontaneous feedback was good from both the BA and the PS groups. Regular group participation and a relatively low rate of dropouts support the view of reasonable patient satisfaction. Sixth, due to the official Finnish translation of the DSM-5 still pending in 2016, we had to use DSM-IV measures. Finally, the study interventions and data collection were conducted in 2016–2017. However, were are not aware of any factors that would significantly undermine the generalizability of findings to current conditions.

The aim of the study was to investigate the treatment of acutely ill depressive patients. However, despite deliberately excluding chronic depression, the recruited patients’ preceding durations of depression were longer than expected. Patients in the study fulfilled the inclusion criteria, but due to delays in treatment onset, the median duration of participants’ treatments within the services before onset was 167 days. Given the preceding phases of treatment-seeking and referral to psychiatric care, the actual mean total duration of their depressive episodes was likely even months longer. Brief BA may not have been an appropriate intervention for psychiatric patients with long-lasting depression and psychiatric comorbidity. Thus, the lack of change in depression scores in the PHQ-9 may be explained at least in part by more chronic depression in patients than initially expected (34). Moreover, long delays in the onset of psychotherapy are known to be adversely associated with efficacy (35; see their figure). However, it is noteworthy that the preceding time in treatment did not correlate with treatment outcomes within the entire sample. Many patients also had comorbid mental disorders, which may have impacted the benefit of group interventions. We did not systematically evaluate psychiatric comorbidity, and thus, the role of comorbidity in the outcome remains unknown. Finally, chance variation may have played some role in our results, as the sample sizes in the treatment arms were relatively small, and the initially least-benefitting BA arm came out as the numerically (but non-significantly) most benefitting one in PHQ-9 re-assessment at the 6-month follow-up (**Table 3**).

We chose the total number of 90-minute BA sessions to be eight in our trial because it was perceived as feasible in psychiatric outpatient care settings. Eight sessions was also the median number of sessions in a meta-analysis of 19 studies of group BA, with the range varying widely between two and 12 sessions (5). It is also a typical duration of treatment in clinical trials of cognitive behavioral therapies overall (8). Thus, the duration of treatment was fully in line with previous research. However, patients’ needs may differ based on their clinical and sociodemographic characteristics. For comparison, in the COBRA multicenter trial (7), BA was provided as individual therapy and included 16 sessions. All core components were delivered by session 8, which they considered to represent a minimally sufficient therapy dose in individual treatment (7). Nevertheless, considering patient characteristics, particularly the long preceding duration of depression, our intervention may have been too brief.

The BA therapists were all experienced psychiatric professionals but were inexperienced with BA before the study. In retrospect, all may not have achieved sufficient competence in BA, nor was this formally tested. The therapists received a brief 8-hour training and 8 hours of clinical supervision in BA during the study. According to video ratings, the therapists varied in how well they implemented the BA manual. Adherence to the manual varied, with scores ranging from 1 to 6, and only half of the videos (six of 12 group sessions) were assessed as suitable or better (≥4). Information from the recorded sessions and about the quality of the treatment was not available for direct feedback to the group leaders during the ongoing interventions, and therefore, the therapists’ actions went uncorrected. From a methodological point of view, our findings demonstrate the importance of critically evaluating adherence to the treatment provided. From a more general view of the scalability of psychosocial interventions, the results highlight potential pitfalls related to the type and duration of training and the quality of treatments being implemented.

The duration of the PS group was also 8 weeks. This group had two purposes in this study: 1) to control specific treatment effects and 2) to assess whether it is helpful in the treatment of depression. However, the treatment consisting of the PS group plus TAU was found to actually be less effective than TAU alone at 8 weeks. The groups were facilitated by an expert with experience in having recovered from depression, and each group included a psychiatric nurse from the outpatient clinic. The presence of a professional was considered necessary to deal with any unexpected situations among group members. The PS group participants found the groups helpful, and the participants gave positive feedback, even though satisfaction was not measured. Group dropout was insignificant. These observations support the paradoxical notion that the groups were considered meaningful, although no positive added influence on the outcome of depression could be demonstrated.

## Conclusion

Despite consistent evidence for the effectiveness of BA overall, in this randomized trial, we did not find the effectiveness of added treatments, comprising the 8-week BA and PS groups, to exceed the usual treatment alone. Whether brief group-format BA provided by briefly trained therapists is suitable for long-term depressive patients in psychiatric settings warrants critical investigation. Applicability of intervention, appropriate training of therapists, and necessary measures to guarantee the quality of treatment should serve as the primary focus points.

## Data availability statement

Data not available due to restrictions imposed by research permit and legislation. Requests to access the datasets should be directed to erkki.isometsa@hus.fi.

## Ethics statement

The studies involving humans were approved by Ethical Committee of the Helsinki and Uusimaa Hospital District. The studies were conducted in accordance with the local legislation and institutional requirements. The participants provided their written informed consent to participate in this study.

## Author contributions

RH: Writing – original draft, Investigation, Formal Analysis, Data curation. TR: Writing – review & editing, Methodology, Formal Analysis, Data curation. LP: Writing – review & editing, Supervision, Methodology, Investigation, Conceptualization. MT: Writing – review & editing, Supervision, Methodology, Conceptualization. EI: Resources, Project administration, Funding acquisition, Writing – review & editing, Supervision, Methodology, Conceptualization.
